# Supplementary material for: Predicting visual function by interpreting a neuronal wiring diagram
Source: Nature. 2024 Oct 2;634(8032):113–23. doi: 10.1038/s41586-024-07953-5 (PMC11446822; doi:10.1038/s41586-024-07953-5)
Supplement: Supplementary file 6 — CRF and ERF predictions for individual TmY4 and TmY9 cells. Analogous to Supplementary Data 3, but for TmY target types. Shown are the top four monosynaptic pathways, the strongest pathway passing through each of the top ten intermediary types (ranking from Extended Data Fig. 7), and the trisynaptic pathway Tm1–TmY–Dm3–TmY (see the section entitled Prediction of spatial normalization). [file 41586_2024_7953_MOESM6_ESM.zip › DataS4/TmY9qΓèÑ/720575940620525377.pdf]

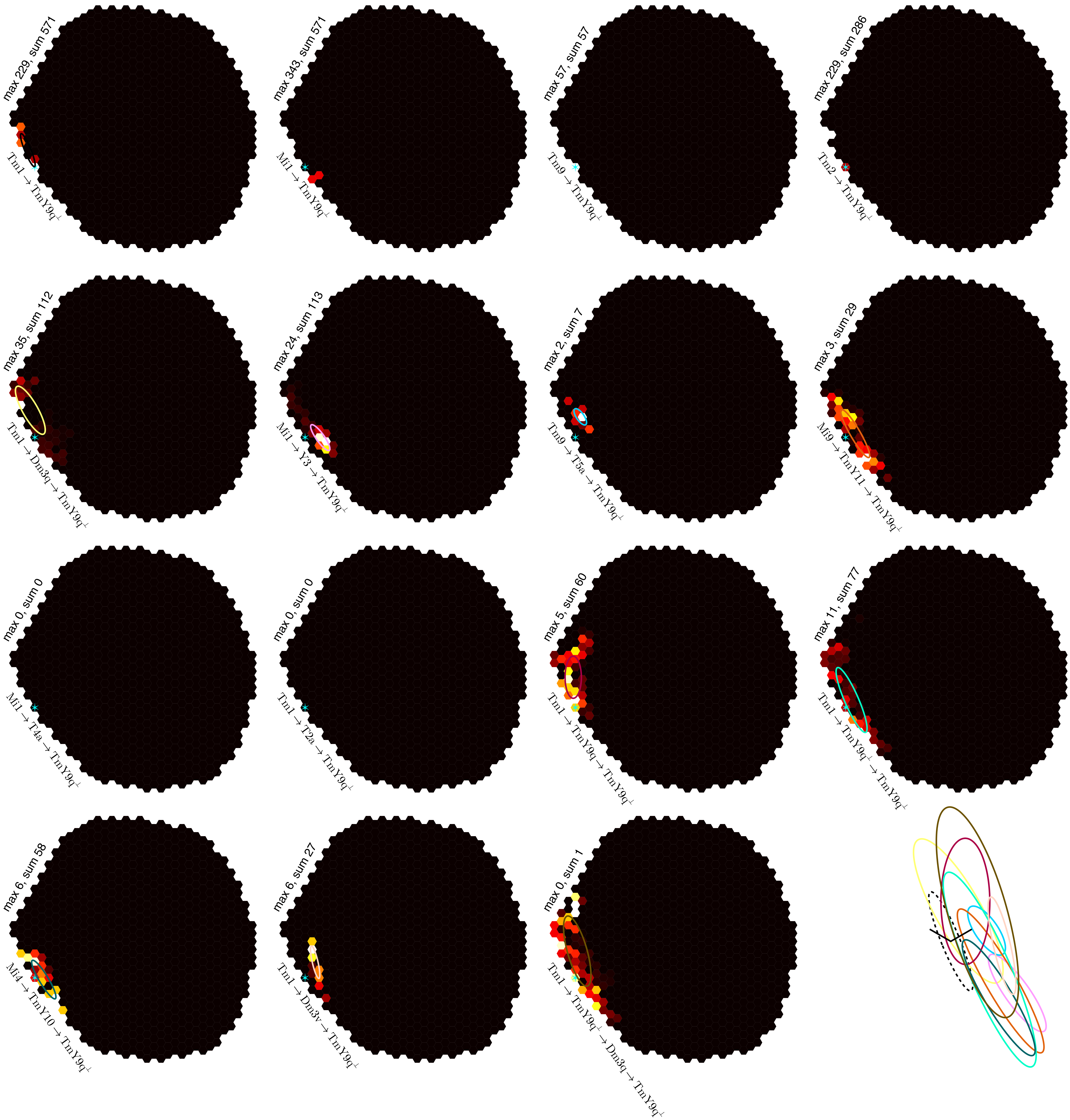
max 229, sum 571
Tm1 → TmY9q⊥
max 343, sum 571
Mi1 → TmY9q⊥
max 57, sum 57
Tm9 → TmY9q⊥
max 229, sum 286
Tm2 → TmY9q⊥
max 35, sum 112
Tm1 → Dm3q → TmY9q⊥
max 24, sum 113
Mi1 → Y3 → TmY9q⊥
max 2, sum 7
Tm9 → T5a → TmY9q⊥
max 3, sum 29
Mi9 → TmY11 → TmY9q⊥
max 0, sum 0
Mi1 → T4a → TmY9q⊥
max 0, sum 0
Tm1 → T2a → TmY9q⊥
max 5, sum 60
Tm1 → TmY9q → TmY9q⊥
max 11, sum 77
Tm1 → TmY9q⊥ → TmY9q⊥
max 6, sum 58
Mi4 → TmY10 → TmY9q⊥
max 6, sum 27
Tm1 → Dm3v → TmY9q⊥
max 0, sum 1
Tm1 → TmY9q⊥ → Dm3q → TmY9q⊥
